# Editorial: Secretion of Cytokines and Chemokines by Innate Immune Cells

**DOI:** 10.3389/fimmu.2015.00190

**Published:** 2015-04-22

**Authors:** Paige Lacy

**Affiliations:** ^1^Pulmonary Research Group, Departments of Medicine and Medical Microbiology and Immunology, University of Alberta, Edmonton, AB, Canada

**Keywords:** SNAREs, macrophages, neutrophils, eosinophils, dendritic cells, epithelial cells, exocytosis, degranulation

Cytokines and chemokines are released from a wide range of immune cells, and are essential for the communication of signals in both innate and adaptive immunity. While there is a substantial body of literature regarding the function of cytokines and chemokines in immunity, remarkably little has been done to determine how these are packaged and secreted from a range of immune cells. Our knowledge of how cells package and release cytokines, chemokines, and other immunomodulatory factors is limited and only just emerging. Understanding how innate immune cells release cytokines and chemokines is important, as these factors are indispensable for communication with other immune as well as non-immune cells for the coordination of inflammatory responses. Moreover, cytokine and chemokine release by innate immune cells is a fundamental mechanism for cross-talk with the adaptive immune system.

Protein trafficking and secretion through both constitutive and regulated exocytosis are well described from many cell types. For this reason, many researchers assume that immune cells secrete cytokines and chemokines through the canonical endoplasmic reticulum (ER) and Golgi pathway before being transported to the cell surface via simple, unidirectional secretory vesicles. However, the reality is much more complex than this. Immune cells utilize a myriad assortment of secretory pathways, involving a combination of secretory granules and vesicles, for trafficking cytokines and chemokines. Perhaps counterintuitively, the secretion of cytokines and chemokines is often associated with endocytic pathways that are engaged in retrograde transport of proteins to the cell interior. The trafficking of cytokines and chemokines occurs through distinct vesicular pathways in each cell type, which adds further complexity to our understanding of this process. In this Research Topic, we will review the ability of a group of innate immune cells to synthesize and secrete various cytokines and chemokines. These are macrophages, neutrophils, mast cells, eosinophils, dendritic cells, and epithelial cells.

Submissions in this Research Topic include reviews that delve into some of the mechanisms described for their release. We begin with macrophages, which are best characterized for their ability to transcribe, translate, and process cytokines and chemokines for release (1, 2). Macrophages lack secretory granules and instead rely on a constitutive secretory pathway that engages recycling endosomes for the secretion of cytokines, including tumor necrosis factor (TNF), interleukin-6 (IL-6), and IL-10. The trafficking machinery required for the secretion of cytokines include soluble NSF attachment protein receptors (SNAREs) and accessory proteins that facilitate membrane fusion, such as GTPases, Munc13, and others. Stimulation of macrophages with the bacterial lipopolysaccharide (LPS) results in *de novo* synthesis of the transmembrane TNF precursor, which is transported through ER and Golgi to be trafficked via recycling endosomes to the cell surface by SNARE-dependent membrane fusion. These steps will be reviewed in detail and set the standard for our understanding of cytokine trafficking in innate immune cells.

Neutrophils will also be reviewed for their ability to release a plethora of cytokines and chemokines (3), which are essential for modulation of immune responses in acute and chronic inflammation. In spite of our knowledge of neutrophil cytokine and chemokine synthesis and secretion, remarkably little is known regarding if or how these are packaged in the neutrophil’s secretory granules, which are well characterized. To explore the secretory capacity of neutrophils, we will review the mechanisms of neutrophil granule protein processing and regulated secretion, and how dysregulation of these mechanisms leads to diseases (4).

In mast cells, we will learn the differential release of granule proteins and the secretory pathways involved (5), and review their ability to regulate post-transcriptional control of cytokine production (6). Mast cell secretion of cytokines occurs through a trafficking mechanism that may resemble that of macrophages. Cytokines are dependent on SNARE-mediated secretion in mast cells, although mast cells appear to utilize different set of SNAREs for vesicular transport of cytokines than that of anaphylactic degranulation.

Eosinophils are major effector cells in allergic inflammation and have the ability to secrete numerous cytokines and chemokines (7). They also have the ability to synthesize and store cytokines for subsequent release. We will review the distinct intracellular membrane trafficking pathways that are used to transport pre-formed cytokines from their intracellular site of storage in eosinophil crystalloid granules to the cell membrane (8). Specifically, we will focus on the secretion of IL-4 and the chemokine CCL5/RANTES through a tubulovesicular transport system associated with piecemeal degranulation, which is also dependent on SNARE-mediated membrane fusion.

Relatively little is known regarding how dendritic cells secrete cytokines and chemokines, and in this Research Topic, we will learn the role of the SNARE syntaxin-3 in the secretion of IL-6 following stimulation of toll-like receptors (9). This will also be reviewed to understand more about how SNAREs may be required for the secretion of cytokines from dendritic cells, and this remains an area of investigation (10).

Finally, we will review the role of secreted cytokines and chemokines from airway epithelial cells and their role in shaping the immune response in allergy (11). The mechanisms of cytokine and chemokine secretion from epithelial cells have not yet been investigated, although this review helps us to understand the importance of epithelium-derived factors in altering immunity.

## Conflict of Interest Statement

The author declares that the research was conducted in the absence of any commercial or financial relationships that could be construed as a potential conflict of interest.
